# Supplementary material for: Bayesian refinement of protein structures and ensembles against SAXS data using molecular dynamics
Source: PLoS Comput Biol. 2017 Oct 18;13(10):e1005800. doi: 10.1371/journal.pcbi.1005800 (PMC5662244; doi:10.1371/journal.pcbi.1005800)
Supplement: S1 Archive — (BZ2) [file pcbi.1005800.s011.tar.bz2 › gromacs_4.62_bayesian_saxs_refi/share/html/online/genbox.html]

genbox


| genbox Main Table of Contents |

**VERSION 4.6  
Sat 19 Jan 2013**

---

### Description

genbox can do one of 3 things:

1) Generate a box of solvent. Specify -cs and -box. Or specify -cs and
-cp with a structure file with a box, but without atoms.

2) Solvate a solute configuration, e.g. a protein, in a bath of solvent
molecules. Specify -cp (solute) and -cs (solvent).
The box specified in the solute coordinate file (-cp) is used,
unless -box is set.
If you want the solute to be centered in the box,
the program editconf has sophisticated options
to change the box dimensions and center the solute.
Solvent molecules are removed from the box where the
distance between any atom of the solute molecule(s) and any atom of
the solvent molecule is less than the sum of the van der Waals radii of
both atoms. A database (vdwradii.dat) of van der Waals radii is
read by the program, and atoms not in the database are
assigned a default distance -vdwd.
Note that this option will also influence the distances between
solvent molecules if they contain atoms that are not in the database.

3) Insert a number (-nmol) of extra molecules (-ci)
at random positions.
The program iterates until nmol molecules
have been inserted in the box. To test whether an insertion is
successful the same van der Waals criterium is used as for removal of
solvent molecules. When no appropriately-sized
holes (holes that can hold an extra molecule) are available, the
program tries for -nmol \* -try times before giving up.
Increase -try if you have several small holes to fill.

If you need to do more than one of the above operations, it can be
best to call genbox separately for each operation, so that
you are sure of the order in which the operations occur.

The default solvent is Simple Point Charge water (SPC), with coordinates
from $GMXLIB/spc216.gro. These coordinates can also be used
for other 3-site water models, since a short equibilibration will remove
the small differences between the models.
Other solvents are also supported, as well as mixed solvents. The
only restriction to solvent types is that a solvent molecule consists
of exactly one residue. The residue information in the coordinate
files is used, and should therefore be more or less consistent.
In practice this means that two subsequent solvent molecules in the
solvent coordinate file should have different residue number.
The box of solute is built by stacking the coordinates read from
the coordinate file. This means that these coordinates should be
equlibrated in periodic boundary conditions to ensure a good
alignment of molecules on the stacking interfaces.
The -maxsol option simply adds only the first -maxsol
solvent molecules and leaves out the rest that would have fitted
into the box. This can create a void that can cause problems later.
Choose your volume wisely.

The program can optionally rotate the solute molecule to align the
longest molecule axis along a box edge. This way the amount of solvent
molecules necessary is reduced.
It should be kept in mind that this only works for
short simulations, as e.g. an alpha-helical peptide in solution can
rotate over 90 degrees, within 500 ps. In general it is therefore
better to make a more or less cubic box.

Setting -shell larger than zero will place a layer of water of
the specified thickness (nm) around the solute. Hint: it is a good
idea to put the protein in the center of a box first (using editconf).

Finally, genbox will optionally remove lines from your topology file in
which a number of solvent molecules is already added, and adds a
line with the total number of solvent molecules in your coordinate file.

### Files

| option | filename | type | description |
| --- | --- | --- | --- |
| **-cp** | protein.gro | Input, Opt. | Structure file: gro g96 pdb tpr etc. |
| **-cs** | spc216.gro | Input, Opt., Lib. | Structure file: gro g96 pdb tpr etc. |
| **-ci** | insert.gro | Input, Opt. | Structure file: gro g96 pdb tpr etc. |
| **-o** | out.gro | Output | Structure file: gro g96 pdb etc. |
| **-p** | topol.top | In/Out, Opt. | Topology file |

### Other options

| option | type | default | description |
| --- | --- | --- | --- |
| **-[no]h** | bool | no | Print help info and quit || **-[no]version** | bool | no | Print version info and quit || **-nice** | int | 19 | Set the nicelevel || **-box** | vector | 0 0 0 | Box size || **-nmol** | int | 0 | Number of extra molecules to insert || **-try** | int | 10 | Try inserting -nmol times -try times || **-seed** | int | 1997 | Random generator seed || **-vdwd** | real | 0.105 | Default van der Waals distance || **-shell** | real | 0 | Thickness of optional water layer around solute || **-maxsol** | int | 0 | Maximum number of solvent molecules to add if they fit in the box. If zero (default) this is ignored || **-[no]vel** | bool | no | Keep velocities from input solute and solvent |

### Known problems

- Molecules must be whole in the initial configurations.

---

http://www.gromacs.org  
gromacs@gromacs.org
